# Synthesis, Characterization of Chitosan-Aluminum Oxide Nanocomposite for Green Synthesis of Annulated Imidazopyrazol Thione Derivatives

**DOI:** 10.3390/polym13071160

**Published:** 2021-04-05

**Authors:** Abir S. Abdel-Naby, Sara Nabil, Sarah Aldulaijan, Ibtisam M. Ababutain, Azzah I. Alghamdi, Somaiah Almubayedh, Khaled D. Khalil

**Affiliations:** 1 Department of Chemistry, College of Science, Imam Abdulrahman Bin Faisal University, P.O. Box 1982, Dammam 31441, Saudi Arabia; snabil@iau.edu.sa (S.N.); saaldulaijan@iau.edu.sa (S.A.); 2 Water Treatment Unit, Basic & Applied Scientific Research Center (BASRC), Imam Abdulrahman Bin Faisal University, P.O. Box 1982, Dammam 31441, Saudi Arabia; iababutain@iau.edu.sa (I.M.A.); azalghamdi@iau.edu.sa (A.I.A.); almubay@uwindsor.ca (S.A.); 3 Department of Biology, College of Science, Imam Abdulrahman Bin Faisal University, P.O. Box 1982, Dammam 31441, Saudi Arabia; 4 Department of Chemistry, Faculty of Science, Cairo University, Giza 12613, Egypt; khd.khalil@yahoo.com; 5 Department of Chemistry, Faculty of Science, Taibah University, Al-Madinah Almunawrah, Yanbu 46423, Saudi Arabia

**Keywords:** chitosan, aluminum oxide, nanocomposite, heterogeneous catalysis, antibacterial activity, molecular docking, imidazopyrazolthione

## Abstract

Chitosan-aluminum oxide nanocomposite was synthesized, characterized, and used as a green heterogeneous catalyst to synthesize novel imidazopyrazolylthione derivatives. Nanocomposite polymeric material was characterized by EDS-SEM and XRD. The powerful catalytic activity, and its base character of the nanocomposite, was used to synthesize imidazopyrazolylthione (**1**) in a good yield compared to traditional cyclocondensation synthesis. Using the nanocomposite catalyst, substitution of the thiol group (**1**) afforded the corresponding thiourea (**2**) and the corresponding ester (**3**). The efficiency of the nanocomposite over the traditional base organic catalyst, Et_3_N and NaOH, makes it an effective, economic, and reproducible nontoxic catalyst. Moreover, the heterogeneous nanocomposite polymeric film was easily isolated from the reaction medium, and recycled up to four times, without a significant loss of its catalytic activity. The newly synthesized derivatives were screened as antibacterial agents and showed high potency. Molecular docking was also performed for a more in-depth investigation. The results of the docking studies have demonstrated that the docked compounds have strong interaction energies with both Gram-positive and Gram-negative bacteria.

## 1. Introduction

Pyrazole and imidazole derivatives are known to exhibit considerable biological activities, such as antiviral [1,2], antimicrobial [3,4] and anti-inflammatory [5]. The synthesis of such heterocyclic compounds required the use of a base catalyst, such as triethylamine or sodium ethoxide. After the Pollution Prevention Act (PPA) in 1990 [6], greener methods attracted the attention of many researchers who were considering the synthesis of heterocyclic compounds [7,8,9]. Among these methods, eco-friendly, nontoxic catalysts and solvents have been suggested [10,11,12,13].

Nanomaterials showed their importance in various scientific fields, such as catalysis and some industrial applications. Accounting for the green approach, some natural biopolymers, such as chitosan and its hybrid materials, have been used as efficient, heterogenous, and recyclable base catalysts for heterocyclic synthesis [14,15]. To increase the base character of chitosan, and to overcome its problem in forming a gel, chitosan-based magnesium oxide and copper oxide nanocomposites were used as efficient catalysts for the regioselective synthesis of (1,2,3) Triazoles [16,17]. On the other hand, metal oxide nanoparticles have found many uses in numerous fields, such as engineering, medicine, drug delivery, agricultural, and nano catalysis applications [18]. Growing interest to explore the utility of alumina as a catalyst, or catalytic support, has also been widely recognized for many organic transformations [19].

In the present work, novel fused imidazopyrazole derivatives have been synthesized via the reaction of pyrazole-5-one with thiourea in the presence of chitosan Al_2_O_3_ nanocomposite as a base catalyst, converting it into imidazo(4,5-c) pyrazole derivatives, and aiming to enhance the biological activity of the newly synthesized derivatives, as they will contain, in their structure, both imidazole and pyrazole moieties.

Moreover, molecular docking, which is a powerful computational tool in drug design, is used to predict the interaction between a ligand and the active site in its receptor [20,21]. In addition, it is used to investigate the best orientation of the ligand binding in the active site. It also scores the strength of the binding energy of the docked ligand with its receptor [22] and thus, it helps us to understand the type of the interactions between the ligand and the receptor. Therefore, the antibacterial potential of the three novel synthesized materials was examined through the determination of their interactions with the active site of their receptor in the bacterial proteins.

## 2. Experimental Section

### 2.1. Materials and Methods

Pyrazole-5(4H)-one, thiourea, ethyl chloroacetate, acetone, ethanol, anhydrous potassium carbonate, aluminum oxide (Al_2_O_3_) nano powder, and chitosan of medium molecular weight (85% deacetylation) were purchased from Sigma Aldrich (St. Louis, MO, USA). Triple distilled water was used in all solution preparation.

Four bacteria strains were used—two gram-positive bacteria (*Staphylococcus aureus* ATCC 29213 and *Staphylococcus epidermidis* ATCC 12228) and two gram-negative bacteria (*Escherichia coli* ATCC25922 and *Pseudomonas aeruginosa* ATCC 27853) obtained from King Fahd Hospital, AlKhobar, Saudi Arabia.

### 2.2. Preparation of Heterogeneous Catalyst (Chitosan-Al_2_O_3_ Hybrid Nanocomposite)

There was 1 g of chitosan (CS), (medium molecular weight; 85% DDA) dissolved in 50 mL of 2% (*v*/*v*) acetic acid solution on a magnetic stirrer (IKA-Werke GmbH & Co, Breisgau-Hochschwarzwald, Germany) for 10 h at room temperature to afford a 2% (*w*/*v*) CS solution. The pH of the resulting CS solution was adjusted to the range of 6–7 by adding the appropriate amount of 1 M NaOH solution under stirring. Now, a suspension of 0.5 g of Al_2_O_3_ (nano powder, <50 nm particle size (TEM, VEGA TS5136MM, TESCAN s.r.o., Brno, Czech Republic), 544833 Sigma-Aldrich) in a small amount of double-distilled water was added portion-wise to the CS solution under continuous stirring. The mixture was further stirred for 3 h at room temperature, then cast into a 100 mm Petri dish, and dried overnight at 70 °C (via an oven-drying (70 °C, BGZ-146, Shanghai Boxun Industry & Commerce CO., Ltd, China) to remove any acetic acid traces. Finally, the obtained CS/Al_2_O_3_ nanocomposite film was detached, washed with distilled water, and dried at 60 °C to ensure that all the solvent removed completely from the film.

### 2.3. Recyclability of the Catalyst

To estimate the appropriate catalyst loading, a model reaction of imidazo pyrazole thione (1) (10 mmol) and choro ethyl acetoacetate (10 mmol) was carried out in 25 mL absolute ethanol using 1, 5, 10, 15, and 20 wt%. of catalyst under the same conditions.

### 2.4. Synthesis of 3-Methyl-1-Phenyl-3a,4-Dihydro Imidazo (4,5-c) Pyrazole-5(1H)-Thione (1) 

Reflux an equimolar quantity (1 mmole) of pyrazol-5(4H) one and thiourea, in presence of catalyst (0.05 mmole), and 30 mL of EtOH for 2 h. Filter on hot and then the excess solvent was evaporated whereupon the precipitation that formed was collected and crystallized from ethanol (Sigma Aldrich (St. Louis, MO, USA). Orange powder (90%), mp.: 125–127 °C.

### 2.5. Synthesis of 1-(3-Methyl-1-Phenyl-1,3a-Dihydroimidazo [4,5-c] Pyrazol-5-yl) Thiourea (2)

Reflux an equimolar quantity of (1) (1 mmole) and thiourea in (30 mL) of DMF, in presence of catalyst (0.05 mmole), reflux the mixture for 16 h, after cooling, pour onto ice, filter, and recrystallized from ethanol (Sigma Aldrich (St. Louis, MO, USA). Orange crystals (85%), mp.: 146–148 °C.

### 2.6. Synthesis of Ethyl 2-((3-Methyl-1-Phenyl-1,3adihydroimidazo [4,5-c] Pyrazol-5-yl) thio) Acetate (3) 

Reflux (1 mmol) of (1) within (1.5 mmol) of ethyl chloroacetate in dry acetone (50 mL) suspended with (3 mmol) anhydrous potassium carbonate in presence of the catalyst (0.05 mmole) for 6 h, in water bath. filter the mixture while hot, wash the product that separated well and crystallized from ethanol (Sigma Aldrich, St. Louis, MO, USA). Brown powder (85%), mp.: 189–191 °C.

### 2.7. Biological Evaluation

#### 2.7.1. In Vitro Antibacterial Evaluation

The antimicrobial activity of three novel imidazopyrazole derivatives were evaluated qualitatively by the paper disk diffusion method [23]. The three chemical materials were prepared in different concentrations as follows: 0.01, 0.02, 0.03, 0.05, and 0.1 g/mL in dimethyl sulfoxide (DMSO) and tested directly. Paper discs measured 6 mm in diameter were soaked separately with 100 μL of the chemical materials and were left to dry. Bacterial inoculums were prepared from previous cultures in Muller–Hinton broth. The bacterial cell suspension turbidity was adjusted to a 0.5 McFarland standard, which represented approximately 1–2 × 10^8^ colony-forming units (CFU)/mL using a Biomerieux DensiCHEK plus meter device (Missouri, MO, USA). Bacterial inoculums at 0.5 mL were transferred to petri plates under sterilized conditions. Melted Muller–Hinton agar at approximately 50 °C was poured over the inoculums, and to ensure even distribution of the inoculums, the plates were rotated neatly, then cultures were left to harden for 5 min. Then, previously prepared paper discs, saturated with chemicals, were transferred, using sterilized forceps, to the surface of petri plates that contained the bacterial cultures. Gentamicin (CN10µg), aminoglycosides antibiotic was used as the positive control and DMSO was used as the negative control. The petri plates were placed in the refrigerator (General Electric, Medford, MA, USA) to allow diffusion of the chemicals into the agar for an hour. Then the petri plates were incubated at 37 °C for 24 h. After the incubation period ended, the area of inhibition around the discs was measured with a ruler. Three replicants were made to ensure the accuracy of the experiment.

#### 2.7.2. In Silico Protein Preparation and Active Site Prediction

The crystal structures of the target proteins, peptide deformylase (PDF) protein for *Staphylococcus aureus* (1N5N) [24], and *Pseudomonas aeruginosa* (1LQW) [25] and the Transcriptional regulator (TcaR) protein for *Staphylococcus epidermidis* (3KP3) [26] were downloaded from a protein data bank (PDB) database (www.rcsb.org, accessed on 3 January 2021) [27]. Peptide deformylase (PDF) [28,29,30,31] and Transcriptional regulator (TcaR) [26] proteins are very attractive targets for antibacterial drugs discovery.

In order to prepare these protein crystal structures for the docking process, AutoDockTools (ADT) version 1.5.6 (Scripps Research, San Diego, CA, USA) [32] was used. The preparation started by removing any ligands, ions, unwanted chains, and water molecules from the original PDB files. Followed by adding the polar hydrogen atoms and the partial charge of the system. The active sites of the target proteins were identified using the CASTp server [33] and have been obtained from protein data bank (PDB) database. Protein–ligand interaction profiler server [34] was a very helpful tool to identify the active sites as well.

#### 2.7.3. In Silico Ligand Preparation

The structures of the synthesized target imidazopyrazole derivatives (1–3) ligands were drawn by using Chem Sketch (ACD Labs, Toronto, ON, Canada, freeware) version 2.5 [35] and the optimized 3D structure was saved. PyMOL software version 4.2.0 (Schrodinger Inc., New York, NY, USA) [36] was used to check the structures.

#### 2.7.4. Molecular Docking Study

AutoDockTools (ADT) version 1.5.6 [32], at default settings, was used to prepare the ligands and the receptors and saved as pdbqt format. AutoDock Vina server version 1.1.2 [37] was used to perform the docking calculations of the ligands in the active sites of the proteins using Lamarckian Genetic Algorithm. For each ligand, thirty docking runs were tested, and the best binding energy score for each ligand with its receptor were listed. The interaction between the ligands and the proteins were evaluated using PyMol version 4.2.0 [36] and by using Protein-Ligand interaction profiler server [34].

## 3. Results and Discussion

**Scheme 1:**
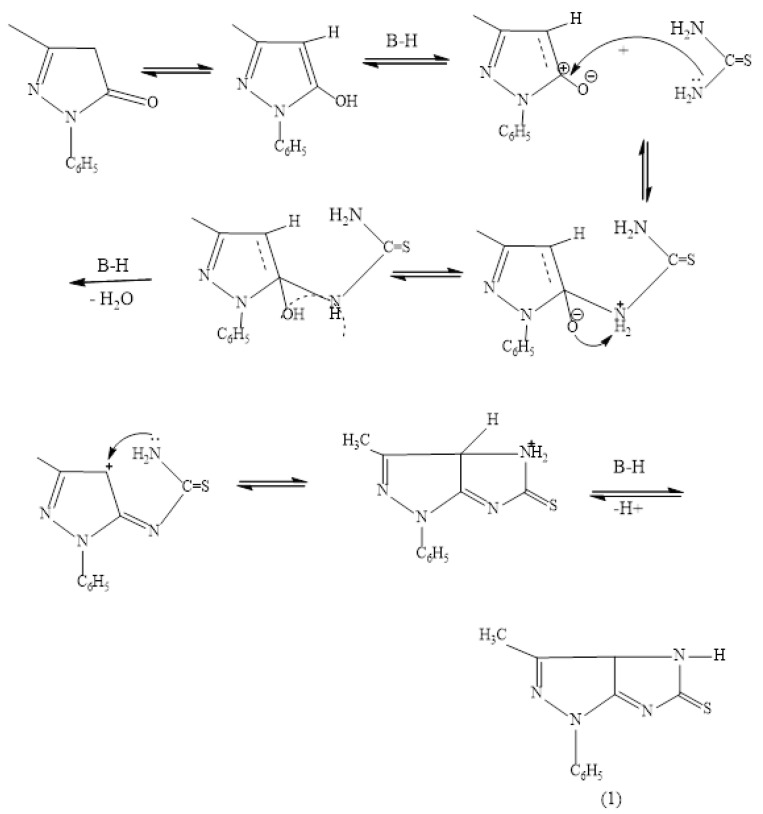
Base catalyzed cyclization of pyrazol-5 (4 H) one (1).

Synthesis of imidazo pyrazoles from the cyclization of pyrazol-5 (4 H) one needs base catalyst, as indicated from the following Scheme 1.

The traditional base catalysts, such as pyridine and triethyl amine, are toxic materials that always gave poor yield since they are homogeneous and hardly get out of the medium to precipitate the product. In the present work, a synthetized polymeric nanocomposite catalyst (CS-Al_2_O_3_) is suggested as the heterogeneous base catalyst.

### 3.1. Preparation of Chitosan-Al_2_O_3_ Hybrid Nanocomposite 

The chitosan-alumina (CS-Al_2_O_3_) nanocomposite was prepared by the simple solution casting method, as indicated in the experimental section. A schematic presentation of chitosan nanomaterial is shown in Scheme 1.

### 3.2. Characterization of Chitosan Based Al_2_O_3_ Nanocomposite

#### 3.2.1. FTIR Characterization

**Figure 1:**
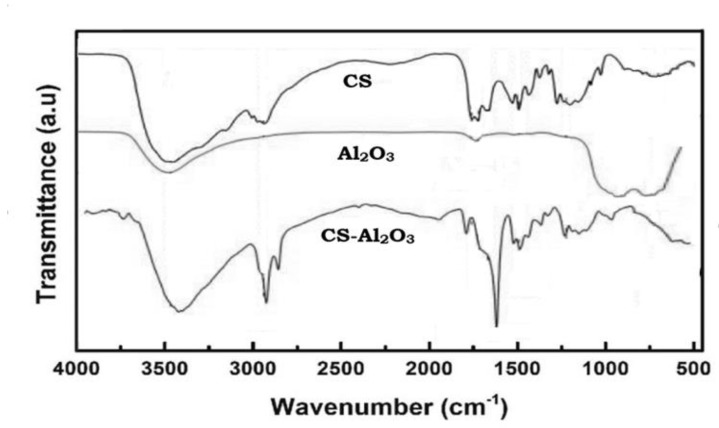
FTIR of chitosan (CS) and Al_2_O_3_ as compared to Chitosan−Al_2_O_3_ nanocomposites.

IR spectra of chitosan and chitosan-Al_2_O_3_ nanocomposites are shown in Figure 1. FTIR spectrum of pure CS revealed a broad O–H stretching band at υ = 3419 cm^−1^, caused by intermolecular H– bonding, that is overlapped with an N–H stretching band in the same region. The characteristic bands for amide groups are found in this spectrum at υ = 3446, 1653, and 1609 cm^−1^ while the bands at 2918, 2875, 1425, and 1380 cm^−1^ in the spectrum assigned to the C–H bonds in CS chain [38]. In Figure 1, the splitting of the broad band above υ = 3000 cm^−1,^ and the appearance of two characteristic bands at 2151 and 2356 cm^−1^ is considered as evidence of the coordination between Al_2_O_3_ and chitosan backbone. Moreover, the interaction between Al_2_O_3_ molecules within the chitosan matrix is clearly viewed by the obvious change in the chitosan fingerprint region.

#### 3.2.2. XRD Characterization

**Figure 2:**
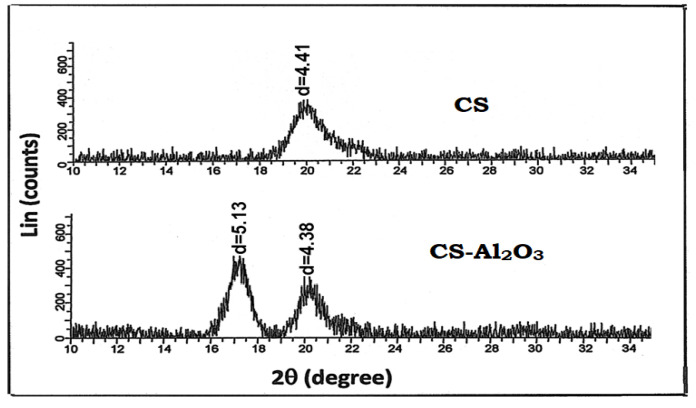
XRD of chitosan−Al_2_O_3_ nanocomposite as compared to the native chitosan.

XRD patterns for chitosan and chitosan-Al_2_O_3_ nanocomposites are shown in Figure 2. The unmodified chitosan (CS) showed the main characteristic peak i.e., one as a strong broad reflection at 2θ (20°–21°), all of which are characteristic of the polymer hydrated crystalline structure [39]. On the other hand, the interaction of chitosan with Al_2_O_3_ resulted in the appearance of an additional strong characteristic peak at 17°, which indicated clear evidence of the interaction between chitosan backbone and Al_2_O_3_ molecules.

#### 3.2.3. Emission Scanning Electron Microscopy (ESEM) and Morphological Changes

**Figure 3:**
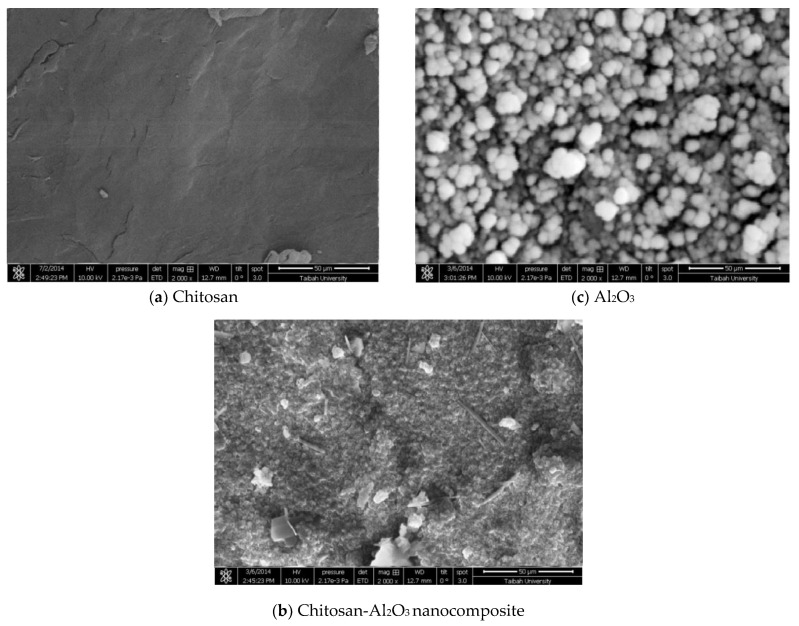
Emission Scanning Electron Microscopy (ESEM) of (**a**): chitosan and (**b**): chitosan-Al_2_O_3_ nanocomposites as compared to that of Al_2_O_3_ (**c**).

A comparison of the surface morphology of unmodified chitosan and chitosan-Al_2_O_3_ nanocomposites is shown in Figure 3. In Figure 3a, the chitosan showed its characteristic fibrous surface while in the chitosan-Al_2_O_3_ nanocomposites. Figure 3b indicated a great change in the surface morphology by the interaction with Al_2_O_3_ nanoparticles. The morphology of Al_2_O_3_ is given for comparison (Figure 3c).

#### 3.2.4. Energy Dispersive X-ray Measurements and Estimation of Aluminum

**Figure 4:**
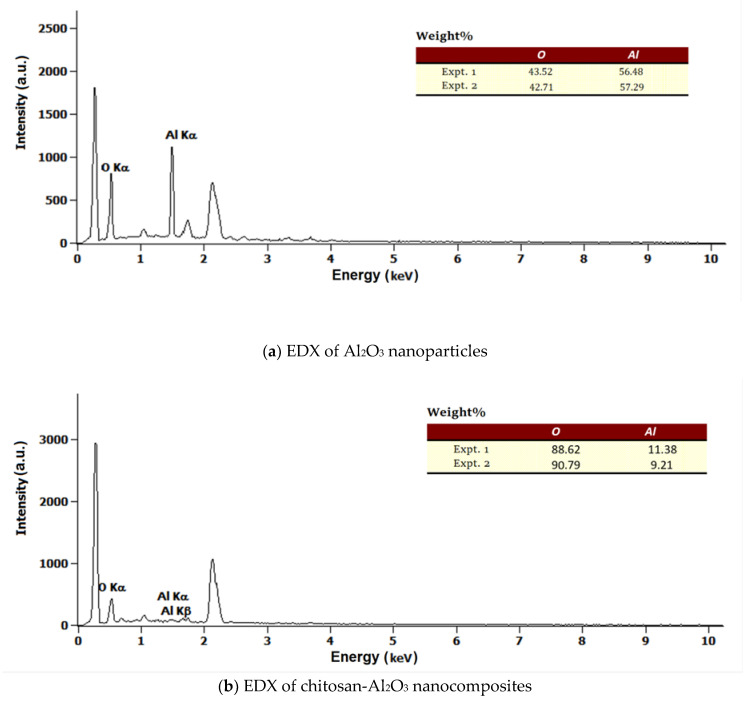
EDS of chitosan- Al_2_O_3_ nanocomposites (**b**) as compared to that of Al_2_O_3_ (**a**).

Energy dispersive X-ray measurements (EDX) are shown in Figure 4, and a comparison of Al_2_O_3_ and chitosan-Al_2_O_3_ nanocomposites by EDX graphs showed the appearance of new signals, related to the incorporated amount of aluminum, in the chitosan backbone. From the EDX results, the elemental analysis of modified composite determined the Al_2_O_3_ content in the matrix to be 10 wt%.

### 3.3. CS-Al_2_O_3_ Nanocomposites as Base Heterogeneous Catalyst for the Synthesis of Imidazo Pyrazolyl Thione Derivatives

**Scheme 2:**
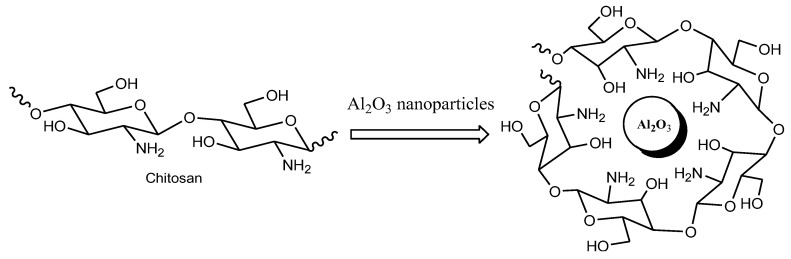
Simplified view of a chitosan-alumina nanocomposite.

One of the difficulties in using native chitosan as a base catalyst in organic syntheses is that separating the product from the polymer is a great challenge. This difficulty is due to the swelling properties of chitosan as it forms a gel. In contrast, the metal-oxide chitosan nanocomposite has more base character by synergistic action, and it does not form a gel during workup of the investigated reactions and, as a result, is a superior catalyst for these reactions. Accordingly, the nanocatalyst can be readily separated by simple filtration, reused after it is washed with hot methanol, and dried in an oven at 100 °C for 2 h more than three times without loss of catalytic activity. Treating pyrazol-5(4H)-one with thiourea in presence of the CS-Al_2_O_3_ nanocomposites, as a base catalyst. The reaction was believed to occur via tautomerization of CH_2_CO into the enolate form, followed by the removal of water molecules and cyclization under the effect of the base catalyst, which dehydrates the cyclized product, affording dihydroimidazo(4,5-c) pyrazole-5(1H)-thione (1) (Scheme 2), in a good yield about 1.4 fold the traditional methods. Moreover, the reaction time was reduced to only 2 h monitoring the product formation with TLC (see Supplementary Materials, Table S1). The formation of this compound was elucidated via IR spectrum (see Supplementary Materials, Figure S1), which revealed absorption bands in the region 3430 cm^−1^ corresponding to NH, 11,600 cm^−1^ due to C=N, and 1252 cm^−1^ attributable for C=S stretching.

On the other hand, the nucleophilic substation starting with thiourea afforded the thiourea derivative (2) in a highly pure form, and in a good yield, due to the basicity of the polymeric catalyst. The product (2) is confirmed by its IR spectrum, which revealed absorption bands in the region 3430, 3350 cm^−1^ corresponding to NH_2_, 11,600 cm^−1^ to C=N, and 1240 cm^−1^ attributable for C=S stretching.

**Scheme 3:**
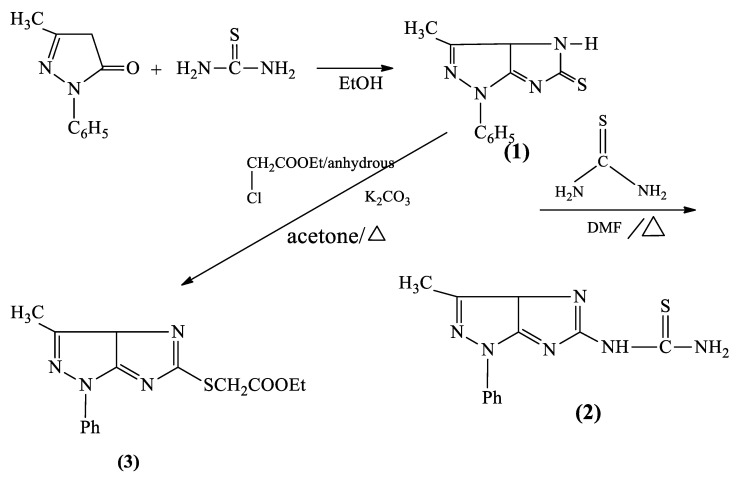
Synthesis of various imidazo pyrazolyl thione derivatives (1), (2), and (3).

The alkylation of imidazopyrazole moiety with ethyl chloroacetate afforded the corresponding ester (3) (Scheme 3). The formation of this ester was obtained in a good crystalline yield, under the basic efficiency of the catalysis, in comparison to the conventional methods. The formation of ester was elucidated via IR spectrum which revealed the absorption bands at 1740 cm^−1^ (υ C=O ester), 1220 cm^−1^ (υ C–O), and the absence of C=S stretching band.

### 3.4. Reusability of the Heterogeneous Chitosan-Aluminum Oxide Nanocomposite for Synthesis of Various Imidazo Pyrazolyl Thione Derivatives

**Figure 5:**
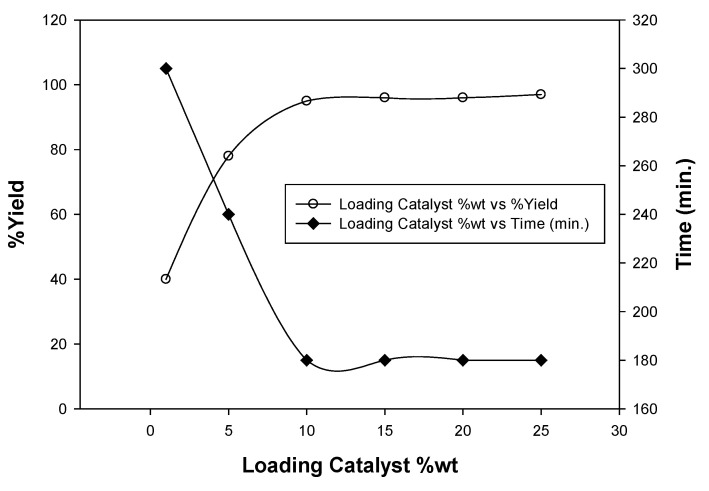
Catalyst loading optimization of the chitosan-aluminum oxide nanocomposite.

To estimate the appropriate catalyst loading, a model reaction of (1) (10 mmol) and choroethyl acetoacetate (10 mmol) was carried out in 25 mL absolute ethanol using 1, 5, 10, 15, and 20 wt%. of catalyst under the same conditions (Figure 5).

The catalyst loading 10 wt% was found to be the optimal quantity for the investigated reaction. Moreover, the catalyst was reused four times, and the results showed that it can be reused as such without significant loss in its catalytic activity (Table 1).

### 3.5. Antibacterial Activities

The antimicrobial activity of the three derivatives was evaluated qualitatively by the disk diffusion method (CLSI-M02-A11–2012). The results are listed in Table 2.

All derivatives exhibited a broad-spectrum activity, as they were able to inhibit both Gram-positive and Gram-negative bacteria with moderate to high inhibition zone range between 7.3 ± 0.6 and 18.7 ± 0.6. The inhibitory activity correlated with the increase in their concentrations however, the low concentrations (0.01, 0.02, 0.03 g/mL) were not able to inhibit the bacterial growth. Although, the area of inhibition zone was less than that of positive control gentamicin (CN10µg), except for (2), its effect was equal to gentamicin on *Staph. aureus* ATCC 29213 at the concentrations 0.05 g/mL and 0.01 g/mL.

### 3.6. Molecular Docking

In silico molecular docking, calculations show that the synthesized imidazopyrazole derivatives ligands have very good interaction energies with the protein structure of both Gram-positive and Gram-negative bacteria. The docking study of the ligands, binding with the target proteins, show very good binding affinity energies for all the three ligands ranging between (−7.06 and −8.68 kcal/mol) (Table 3).

#### 3.6.1. Pseudomonas Aeruginosa

**Figure 6:**
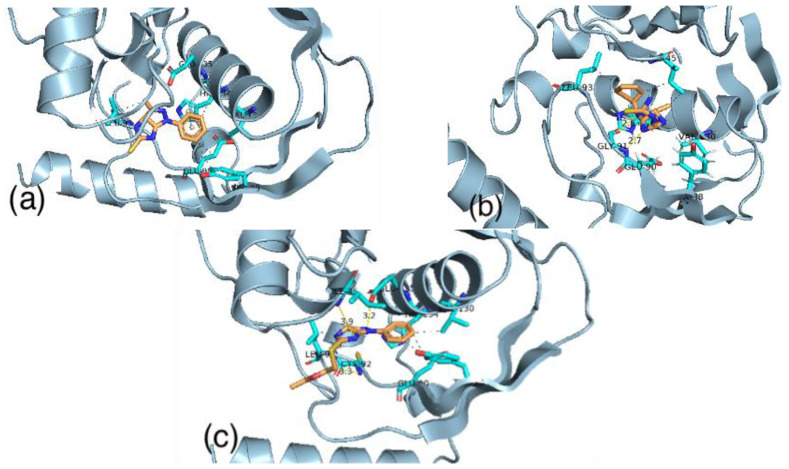
The binding of ligand (1) (**a**) ligand (2) (**b**) and ligand (3) (**c**) with the active site of *Pseudomonas aeruginosa.*

Imidazo(4,5-c)pyrazole5thione (**1**) ligand has a binding score equal to −7.73 kcal/mol. This ligand binds with the active site by stacking interactions with HIS134 and by hydrophobic interactions with TYR88, GLU90, LEU93, VAL 130 and HIS134 in the active site of the receptor. On the other hand, the thiourea derivative (**2**) interacts with the active site by hydrogen bonds with GLY91 and by hydrophobic interactions with ILE45, TYR88, GLU90, LEU93, HIS134, and GLU135. These interactions give a total binding energy equal to −8.17 kcal/mol. We believe that the hydrogen bonds between this ligand and the active site have a stabilization effect for this complex. Meanwhile, the imidazopyrazole ester (**3**)**,** which has the highest binding score with a receptor among all the calculations (−8.68 kcal/mol), interacts with the active site by four hydrogen bonds with CYS92, LEU93, ILE35, and GLU135. In addition, it also has hydrophobic interactions with TYR88, GLU90, LEU93, VAL 130, and HIS134 in the active site of the receptor (Figure 6).

#### 3.6.2. Staphylococcus Aureus

**Figure 7:**
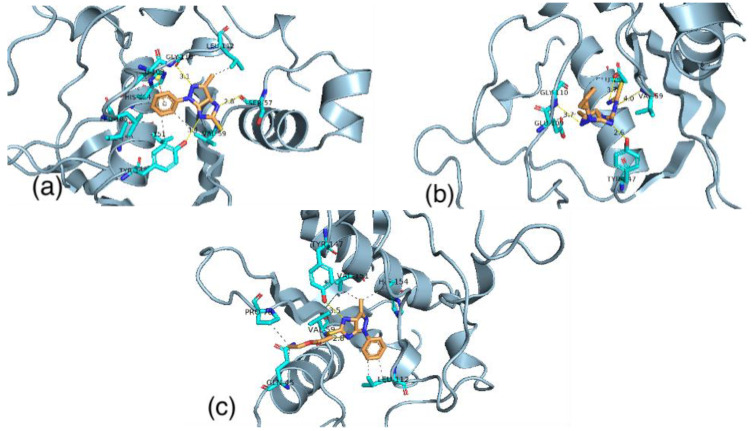
The binding of ligand (**1**) (**a**) ligand (**2**) (**b**) and ligand (**3**) (**c**) with the active site of *Staphylococcus aureus.*

Imidazopyrazolethione ligand (**1**) has a binding energy equal to −7.11 kcal/mol. The binding of this ligand is controlled by hydrogen bonds with SER57, GLY110, and TYR147. In addition, it is controlled by the hydrophobic interactions with VAL59, LEU105, GLU109, LEU112, ILE150, and VAL151 of the active site of the receptor. The thiourea derivative (**2**) interacts with the active site by hydrogen bonds with VAL59, GLY110, TYR 147, and GLU155. Moreover, this binding of the ligand is managed by hydrophobic interactions with GLU109. These interactions give a total binding energy equal to −8.12 kcal/mol). The imidazopyrazol ester (**3**) has the binding score with the receptor equal to −7.98 kcal/mol. This ligand interacts with the active site by hydrogen bonds with VAL59 and TYR147. In addition, it also has hydrophobic interactions with GLN45, PRO78, LEU112, VAL151, and HIS154 in the active site of the receptor (Figure 7).

#### 3.6.3. Staphylococcus Epidermidis

**Figure 8:**
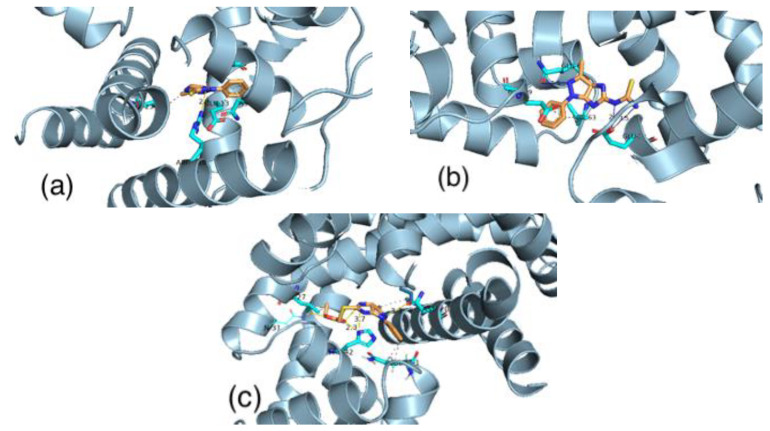
The binding of ligand (1) (**a**) ligand (2) (**b**) and ligand (3) (**c**) with the active site of *Staphylococcus epidermidis.*

Imidazopyrazolethione (**1**) ligand has a binding energy equal to −7.06 kcal/mol. The binding of this ligand is controlled by hydrogen bonds with ARG110A. In addition, it controlled by the hydrophobic interactions with GLU13B, THR23A, ASN45A, and HIS42A of the active site of the receptor. The thiourea derivative (**2**) interacts with the active site by hydrogen bonds with GLU39A and GLN61B. Moreover, this binding of the ligand is controlled by hydrophobic interactions with GLU30B and VAL63A. These interactions give a total binding energy equal to −7.89 kcal/mol. The imidazopyrazol ester (**3**) has the binding score with the receptor equal to −7.67 kcal/mol. This ligand interacts with the active site by hydrogen bonds with GLN31A, ASN20A, and HIS42A. Moreover, it also has hydrophobic interactions with GLN61A, LEU27A, and ILE16B in the active site of the receptor (Figure 8).

## 4. Conclusions

(1) In this work, hybrid CS/Al_2_O_3_ nanocomposite film was successfully prepared via the simple solution casting method, and the structural properties of obtained nanocomposite film were studied carefully by using FTIR, SEM, EDX, and XRD tools. This hybrid nanocomposite showed an obvious catalytic potency for synthesis of annulated imidazopyrazolthione derivatives with an excellent yield. The optimal catalyst loading was 10 wt%, and the catalyst could be easily removed from the reaction mixture and effectively reused three times without significant loss of its catalytic efficiency.

(2) The unique capability of the polymeric catalysis had a noticeable effect in the synthesis of three imidazopyrazole derivatives, taking the advantage of mining preparation time and obtaining a highly percentage of crystalline. These derivatives were examined as antibacterial agents and showed remarkable activity, which is in agreement with the in-silico studies.

(3) The result of the molecular docking study shows that the imidazopyrazole ligands have the capability to bind with the active site of the target proteins for both Gram-positive and Gram-negative bacteria. Thus, we believe that these compounds are promising candidates for antibacterial drugs.

## Figures and Tables

**Table 1:** Recyclability of the chitosan-aluminum oxide nanocomposite as base catalyst.

Scheme 1	Fresh Catalyst	Recycled (1)	Recycled (2)	Recycled (3)	Recycled (4)
Product 2 (%Yield)	95	94	93	92	92

**Table 2:** Antibacterial activity of the three derivatives, at different concentrations using paper disk diffusion method, zone of inhibition (mm) means ± SD *.

Bacterial Test	(1)		(2)		(3)		Positive Control Gentamicin (CN10 µg)
	0.05 g/mL	0.1 g/mL	0.05 g/mL	0.1 g/mL	0.05 g/mL	0.1 g/mL	
*P. aeruginosa* ATCC 27853	10.0 ± 1.0	11.0 ± 1.0	7.7 ± 0.6	8.3 ± 0.6	7.3 ± 0.6	7.3 ± 0.6	20.7 ± 1.2
*Staph. epidermidis* ATCC 12228	8.3 ± 0.6	8.3 ± 0.6	8.3 ± 0.6	9.3 ± 0.6	7.3 ± 0.6	8.7 ± 0.6	21.3 ± 1.2
*Staph. aureus* ATCC 29213	7.7 ± 0.6	9.0 ± 1.0	18.0 ± 0.0	18.7 ± 0.6	7.3 ± 0.6	8.7 ± 0.6	18.7 ± 1.5

* The negative control results (DMSO) were zero and the low concentrations (0.01, 0.02, 0.03 g/mL) of the three derivatives had no antibacterial activity against the tested bacteria. Therefore, it is not mentioned in the table.

**Table 3:** The docking score (interaction energy (Kcal/mol)) for ligands with the active side of the protein.

Bacteria	Ligands		
	(1)	(2)	(3)
*Pseudomonas aeruginosa*	−7.73	−8.17	−8.68
*Staphylococcus aureus*	−7.11	−8.12	−7.98
*Staphylococcus epidermidis*	−7.06	−7.89	−7.67

## Data Availability

The data presented in this study are available on request from the corresponding author.

## Supplementary Materials

The following are available online at https://www.mdpi.com/article/10.3390/polym13071160/s1, Figure S1: FTIR of various imidazo pyrazolyl thione derivatives. Table S1: Following the formation of the derivatives by TLC.
