# Supplementary material for: Synthesis, Characterization of Chitosan-Aluminum Oxide Nanocomposite for Green Synthesis of Annulated Imidazopyrazol Thione Derivatives
Source: Polymers (Basel). 2021 Apr 5;13(7):1160. doi: 10.3390/polym13071160 (PMC8038599; doi:10.3390/polym13071160)
Supplement: Supplementary file 1 [file polymers-13-01160-s001.pdf]

Synthesis, Characterization of Chitosan-Aluminum Oxide Nanocomposite for Green Synthesis of Annulated Imidazopyrazol Thione Derivatives

Abir S. Abdel-Naby [1,2,*], Sara Nabil [1,2], Sarah Aldulaijan [1,2], Ibtisam M. Ababutain [2,3], Azzah I. Alghamdi [2,3] Somaiah Almubayedh [2] and Khaled D. Khalil [4,5]

1 Department of Chemistry, College of Science, Imam Abdulrahman Bin Faisal University, P.O. Box 1982, Dammam 31441, Saudi Arabia; snabil@iau.edu.sa (S.N.); saaldulaijan@iau.edu.sa (S.A.)
2 Water Treatment Unit, Basic & Applied Scientific Research Center (BASRC), Imam Abdulrahman Bin Faisal University, P.O. Box 1982, Dammam 31441, Saudi Arabia; iababutain@iau.edu.sa (I.M.A.); azalghamdi@iau.edu.sa (A.I.A.); almubay@uwindsor.ca (S.A.)
3 Department of Biology, College of Science, Imam Abdulrahman Bin Faisal University, P.O. Box 1982, Dammam 31441, Saudi Arabia
4 Department of Chemistry, Faculty of Science, Cairo University, Giza 12613, Egypt; khd.khalil@yahoo.com
5 Department of Chemistry, Faculty of Science, Taibah University, Al-Madinah Almunawrah, Yanbu 46423, Saudi Arabia
* Correspondence: aabdelnaby@iau.edu.sa

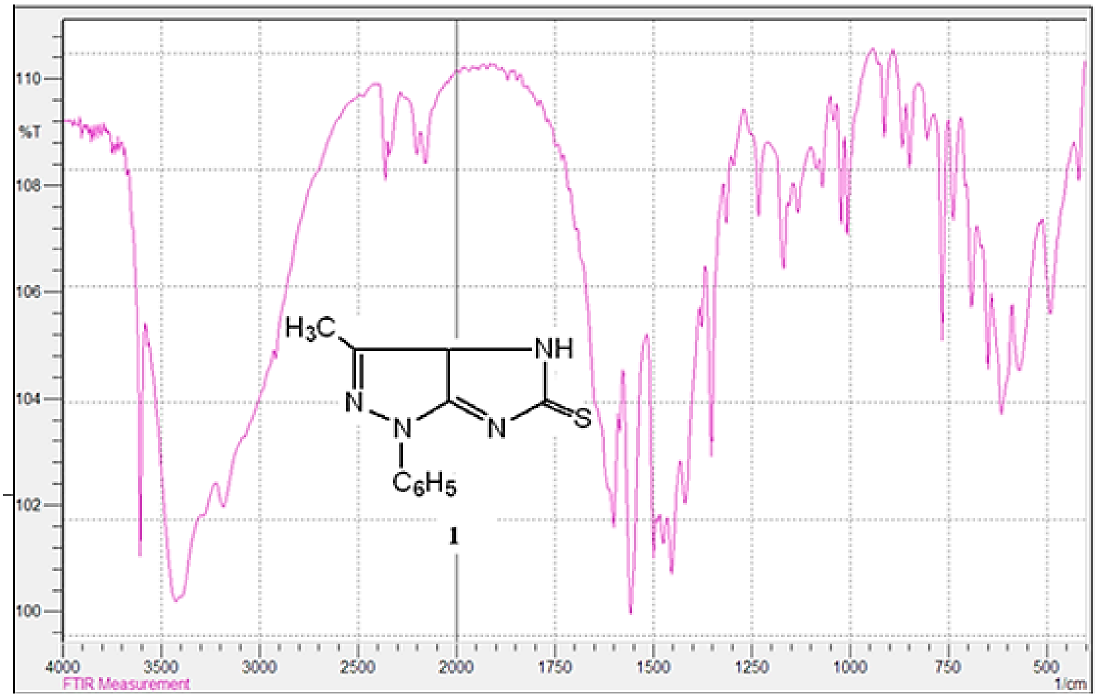

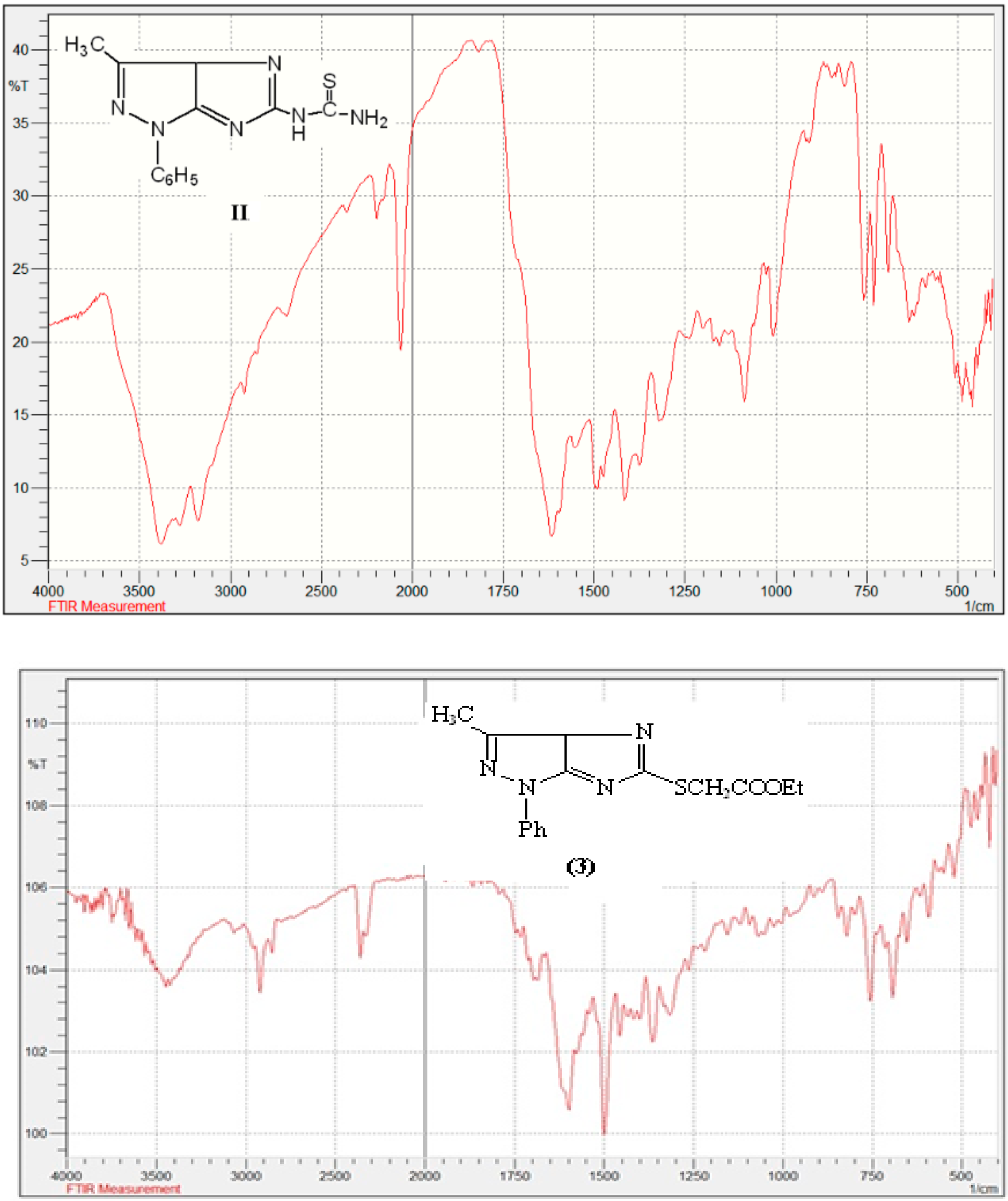

Figure S1. FTIR of various imidazo pyrazolyl thione derivatives.

Table S1. Following the formation of the derivatives by TLC.

Product	Yield	TLC	Reaction time	m.p.
**1**	**90 %**<br>Mass<br>Trial 1 = 0.8 g<br>Trial 2 = 1.2 g	1 : 1<br>Diethyl ether : n-hexane	2 h	125–127 °C /<br>Orange powder
**2**	**85 %**<br>Mass<br>Trial 1 = 0.650 g	1 : 1<br>Diethyl ether : n-hexane<br>(band of SM within the range of product)	18 h	146–148 °C /<br>Orange crystal
**3**	**85 %**<br>Mass<br>Trial 1 = 0.722 g<br>Trial 2 = 0.695 g	1 : 1<br>Diethyl ether : n-hexane	6 h	189–191 °C /<br>Brown powder
